# Detecting robust time-delayed regulation in *Mycobacterium tuberculosis*

**DOI:** 10.1186/1471-2164-10-S3-S28

**Published:** 2009-12-03

**Authors:** Iti Chaturvedi, Jagath C Rajapakse

**Affiliations:** 1 Bioinformatics Research Center, School of Computer Engineering, Nanyang Technological University, Singapore, 639798; 2 Department of Biological Engineering, Massachusetts Institute of Technology, Cambridge, MA, 02139, USA; 3 Singapore-MIT Alliance, Singapore, 117543

## Abstract

**Background:**

Time delays are often found in gene regulation though most techniques of building gene regulatory networks are not capable of capturing such phenomena. Here we look at the delays in the DNA repair system of *Mycobacterium tuberculosis *which is unusually slow in the bacteria. We propose a method based on a skip-chain model to study this phenomena in gene networks. The Viterbi paths of the underlying Markov chains find the most likely regulatory interactions among genes, taking care of very long delays. Using the derived networks, we discuss the delayed regulations and robustness of the DNA damage seen in the bacterium.

**Results:**

We evaluated our method on time-course gene expressions after DNA damage with Mitocyin C. Several time-delayed interactions were observed with our analysis. The presence of hubs in the networks indicates that a small number of transcriptional factors regulate the rest of the system. We demonstrate the use of priors to overcome over-fitting problem in the generation of networks. We compare our results with the gene networks derived with dynamic Bayesian networks (DBN).

**Conclusion:**

Different transcription networks are active at different stages, and constant feedback and regulation is maintained throughout the activities of a biological pathway. Skip-chain models are capable of capturing, long distant and the time-delayed regulations. Use of a Dirichlet prior over parameters and Gibbs prior over structure can greatly reduce the over-fitting in the new model.

## Background

Cellular activities of genes and gene products represented in gene regulatory networks (GRN) provide a basis for signal transduction pathways. Since the signal transduction is transient, the study of dynamics of the transduction is essential. Further, the *distributed *nature of cell fate regulation events manifest's itself as intense crosstalk between the nominal pathways. States of gene networks are often presumed to be stable, meaning that slight changes in the state of a few parents do not change the expression state of the child gene. This phenomena relates to the redundancy of biological systems which are to ensure that the system retains functioning inspite of the perturbations.

In this work, we use Bayesian networks (BN) in the stochastic framework to represent GRN. Pathways have a natural representation of BN, where genes are nodes in the network and edges are causal interactions among them. The causal dependencies are given as conditional probabilities which infer 'cause and effect' relationships among genes in the network. A BN being acyclic is not able to model feedbacks and self-regulation events. The dynamic Bayesian network (DBN) is defined by a pair of structures (*S*_*t*_, *S*_*t*+1_) each corresponding to time instances *t *and *t *+ 1 and a transition network of interactions between the two networks [1]. DBN assumes that the genetic regulation process is first-order Markovian where parents are from the previous time point and can allow cyclic events.

However, several time-delayed interactions are known to exist in biological systems. DBN was extended to a higher-order where mutual information (MI) has been used to determine the best time-delay of an interaction [2]. However, these generative models become intractable at very high orders, so we resort to a conditional skip-chain model. In a skip-chain model, the linear features model the lower-order delays and the skip features model long-distant delays [3].

The linear feature attempts to model interactions which occur instantly or with little delay. The skip feature model interactions occurring much later in the pathway, for example, a gene *g*_*i *_inhibits a gene *g*_*j *_to start a process, and later *g*_*i *_regulates another gene *g*_*k *_towards the end of the process. The skip-feature probability is decomposed into a sum of terms for consecutive pairs of genes in the time-course and the most likely interactions are found using the Viterbi algorithm. The Viterbi skip-feature can automatically determine the best time delay in a higher-order Markov chain representing the instantaneous network of DBN.

Our approach consists of three stages: first, our method involves identifying time-delayed interaction features and predicting the optimal GRN by using a GA. The fitness function of the GA is modified to include Viterbi scores of time-delayed interactions by using the skip-chain model. Next, an application to DNA repair system of *Mycobacterium tuberculosis *has been performed. This bacteria causes tuberculosis in man and is known to have a very slow growth rate *in vitro*. In particular, we consider the DNA repair pathway which is activated when a damage to the DNA occurs. The system consists of proteins *LexA *and *RecA *as well as up to 40 genes that are regulated by these two proteins together. Lastly, we discuss our findings and directions for future work.

## Methods

BN decomposes the joint probability of genes into a product of conditional probabilities by using the chain rule and independence of non-descendant genes, given their parents

where *x *= (*x*_1_, *x*_2_, ...., *x*_*n*_), the conditional probability of gene expression *x*_*i *_given its parents *a*_*i *_is *p*(*x*_*i*_|*a*_*i*_, *θ*_*i*_), and *θ*_*i *_denotes the parameters of the conditional probabilities.

The acyclic condition in BN does not allow self regulation and feedback, which are characteristic of GRN. To overcome this limitation, dynamic Bayesian networks (DBN) are used in which a transition network from one time point to the next characterizes the GRN. The first-order DBN is defined by a transition network of interactions between a pair of structures (*S*_*t*_, *S*_*t*+1_) corresponding to time instances *t *and *t *+ 1. The DBN structure is obtained by unrolling the transition network over time. In time instance *t *+ 1, the parents of genes are those specified in the time instant *t*. The likelihood of transition network *S *of interactions between time instances *t *and *t *+ 1 is given by

where  correspond to the number of instances of *θ*_*ijk *_= *p*(*x*_*i*, *t*+1 _= *k|a*_*i*, *t *_= *j*), *k *is the discretized gene expression level, and *j *is the discrete state combination of parent genes. The first-order DBN has two layers of genes, and therefore 2*n *nodes.

The classical DBN is unable to capture complex time-dependencies and is extended to an *o*-order Markov chain (*o *≥ 2). It predicts the expression levels of a set of genes based on expression upto previous *o *time points. However, such an approach cannot handle long range dependencies because as *o *increases the search space becomes intractable. Instead, we employ skip-chain models which augments linear chain features that represent local features, with skip-features representing long range dependencies [4,5]. It then simply factorizes the prediction probabilities into linear and skip features.

**Figure 1:**
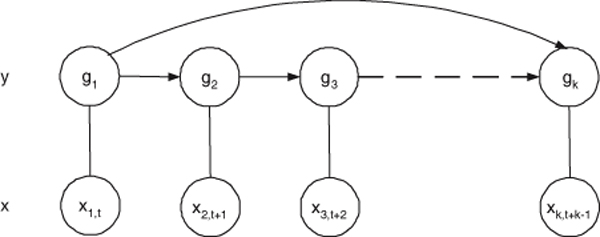
**A skip chain model**. A skip chain model has overlapping skip-edges which model long-distant dependencies.

Linear-chain feature functions *f*(*x*_*i*_, *a*_*i*(*t*-*o*:*t*)_, *t*) represent local dependencies that are consistent with an *o*- order Markov assumption of gene expressions. But for long distant interactions, we relax this assumption by using skip-chain feature functions *h*(*x*_*i*_, *a*_*i*_, *s*_*t*_, *t*) which exploit dependencies between genes that are arbitrarily distant at time instances *s*_*t *_and *t*, respectively (Fig. 1). Such a skip-feature models variable length Markov chain upto *m *- 1 order where *m *is the number of time points.

We can interpolate the two types of features [6]. The log likelihood of an expression *x*_*i *_is a weighted sum of linear and skip-edge scores:

where *λ *≤ 1 is a weight determined heuristically.

For interactions, we look for causal effects of regulated genes as features. We can use the Viterbi algorithm to find a maximum likelihood (ML) path between two genes at distant time points in a hidden Markov model (HMM) [7]. The ML can then be used to make a choice between different time-delayed interactions of the same pair of genes. For any two genes *g*_*i *_and *g*_*j*_, we choose the highest Viterbi score among all the possible interaction features.

A genetic algorithm is used to find the optimal network structure. Here an individual is defined by matrix {*c*_*i*, *j*_}_*n *× *n *_with dimension *n *× *n*. Each cell *c*_*i*, *j *_is randomly initialized with interactions which have MI at a time lag *o *above a threshold. Here *g*_*j *_is the parent of *g*_*i*_. The GA then finds the structure with the highest posterior probability (Eq. 3). The GA provides an optimal structure maximizing the likelihood asymptotically. We also explored the use of two priors over the network.

### Dirichlet prior over parameters

Most higher-order Markov models are far from optimal. They are extremely sensitive to change in pathways and associated data. This happens as most of the data is general rather than feature specific for an interaction. The goal of adaption has been to make good use of available feature data and reduce the over-fitting in the model. Our adaption model combined the reliable general DBN with a volatile feature specific HMM for long delays. We further extend the MLE to a Bayesian learning where a Dirichlet conjugate prior is imposed on each of the parameters.

Given the set of conditional distributions with parameters *θ *= {*θ*_*i*_: *i *= 1, 2, ... *n*}, the likelihood can be written as

The integral can be easily written in a closed form due to conjugacy between Dirichlet and multinomial distribution. However, we can alternatively maximize probability as (MAP):

Using the linear feature as a Dirichlet conjugate prior [8] for the skip feature of a gene we get:

where *h'*(*x*_*i*_) is total probability of the skip-path, *α *is a weighting factor between the linear and skip features.

Next, we can specify the interpolated probability of gene *g*_*i *_based on linear and skip-edges.

here, instead of using a constant, *λ *is estimated using prior linear feature and the total probability of the skip path.

### Gibbs prior over graph

We can use a Gibbs Markov network (MN) to model the prior *P*(*S*) of the gene network. A Gibbs distribution takes the form of *P*(*S*) ∝ *e *- ^*E*(*S*) ^where energy of the graph *E*(*S*) can be factorized into a sum of interaction potentials *U*_*ij *_between genes *g*_*i *_and *g*_*j*_. If an interaction exists in the target network, we set *U*_*ij *_= *σ*_1 _otherwise *U*_*ij *_= *σ*_2_. The total energy of the graph over existing edges is *E*(*S*) = ∑_{*i*, *j*} ∈ *S *_*U*_*ij*_. The posterior probability of the graph is then given by

A small *σ*_1 _and a large *σ*_2 _will reflect the prior target network more in the GRN and vice-versa.

## Experiments and results

We evaluated our method on a DNA repair system of *Mycobacterium tuberculosis *by building regulatory networks with DBN, HDBN, and skip-chain model. Here we looked at the response of bacteria to drug-induced stress. Treatment with Mitomycin C caused DNA damage and hence led to the upregulation of associated repair genes. Eight time points are available at NCBI Gene Expression Omnibus (GSE1642-GPL1396 series) 0.33 hr, 0.75 hr, 1.5 hr, 2 hr, 4 hr, 6 hr, 8 hr and 12 hr after DNA damage. The data was discretized into 1 for upregulation and 0 for downregulation by using an approach described previously [9].

The corresponding skip probabilities were calculated as described in methods. Upto seven time points of delays were allowed. Firstly, we used 9 genes previously specified [10]. In order to get an expanded dataset, the original dataset was subjected to ICA and the components closest to 9 genes were identified [11,12].

This gave us a second dataset of 32 genes. A GA was used to find the optimal structure. Only linear interactions determined by mutual information (MI) upto a time lag of four were allowed. The GA chooses the network with the best combination of skip and linear edges. Simulation was done at different numbers of individuals (N) and generations (G) (N = 200/300/400 and G = 300/400/500) for both HDBN and skip-chain model. The GA stops when the maximum number of generations is reached or if the score does not change for 20 consecutive generations. A similarity threshold of 0.7 in each generation prevents local maxima. The best prediction among all five runs was considered. Table 1 explains the predictions of GRN by using a single time-delay DBN, upto four time delays HDBN, and upto four time delays skip-chain model for both datasets. It can be seen that the ML of the underlying skip-chain prediction is much higher than the DBN or HDBN, confirming that the network fits data well.

**Table 1:** Time-delayed interactions in predicted network

			Higher-order edges				
**# Genes**	**Model:*o***	**ML**	**1**	**2**	**3**	**4**	**5**

9	DBN:1	-14.7	9				
	HDBN:3	-8.69	8	2	7		
	SKIP-CHAIN:1	-6.05	13		(3)		

32	DBN:1	-48.9	36				
	HDBN:4	-39.4	20	6	14	20	
	SKIP-CHAIN:2	-37.2	54	18	(41)	(4)	

Time delayed interactions in predicted DBN, HDBN, and skip-chain: *(n) *denotes overlapping skip-edges and *o *is order of the model.

We also looked at the use of Gibbs prior over the structures, Dirichlet prior over parameters and the combination of the two priors together (Table 2). Using priors further increased likelihood and gave many new time-delayed interactions. Though Dirichlet is a better prior than Gibbs, the combined use of both priors is optimal. Our method also detects many long-time delayed interactions. Some interactions are also observed at order-5 or 6 hrs from the start of the experiment.

**Table 2:** Time-delayed interactions in predicted network using prior

			Higher-order edges				
**# Genes**	**Model:*o***	**ML**	**1**	**2**	**3**	**4**	**5**

9	SKIP-CHAIN:1	-6.05	13		(3)		
	SKIP-CHAIN(Gibbs):1	-5.8	11		(2)		
	SKIP-CHAIN(Dirichlet):2	-5.2	7	13	(11)	(1)	
	SKIP-CHAIN(Gibbs and Dirichlet):3	-3.27	2	7	(4)	(5)	

32	SKIP-CHAIN:2	-37.2	54	18	(41)	(4)	
	SKIP-CHAIN(Gibbs):3	-35.7	37	16	24	(40)	(3)
	SKIP-CHAIN(Dirichlet):2	-35.05	54	16	(37)	(4)	
	SKIP-CHAIN(Gibbs and Dirichlet):2	-34.54	50	15	(41)	(4)	

Time delayed interactions in predicted skip-chain without prior, with Dirichlet prior, with Gibbs prior and combination of both priors: *(n) *denotes overlapping skip-edges and *o *is order of the model.

**Figure 2:**
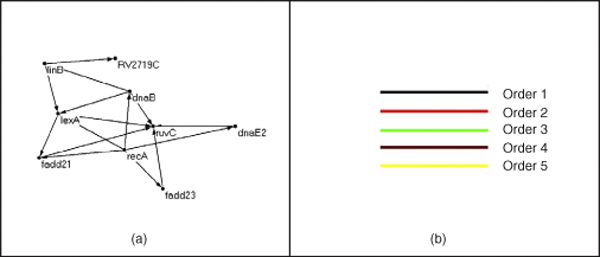
**Target network and color code**. (a) Network determined by correlation and (b) color code.

**Figure 3:**
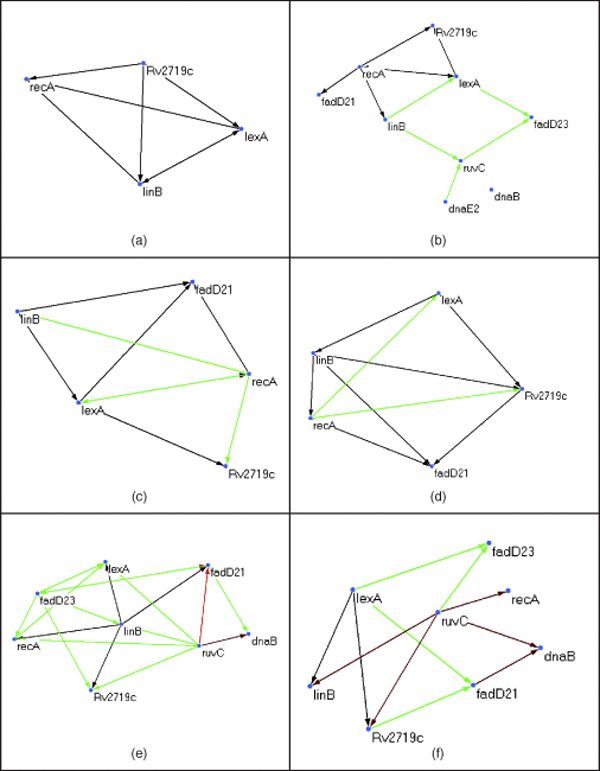
**Time-delayed interactions in predicted network of 9 genes**. Time-delayed interactions in predicted network of 9 genes (a) DBN network, (b) HDBN network, (c) Skip-chain network, (d) Skip-chain network with Gibbs prior, (e) Skip-chain network with Dirichlet prior, (f) Skip-chain network with Gibbs and Dirichlet prior.

**Figure 4:**
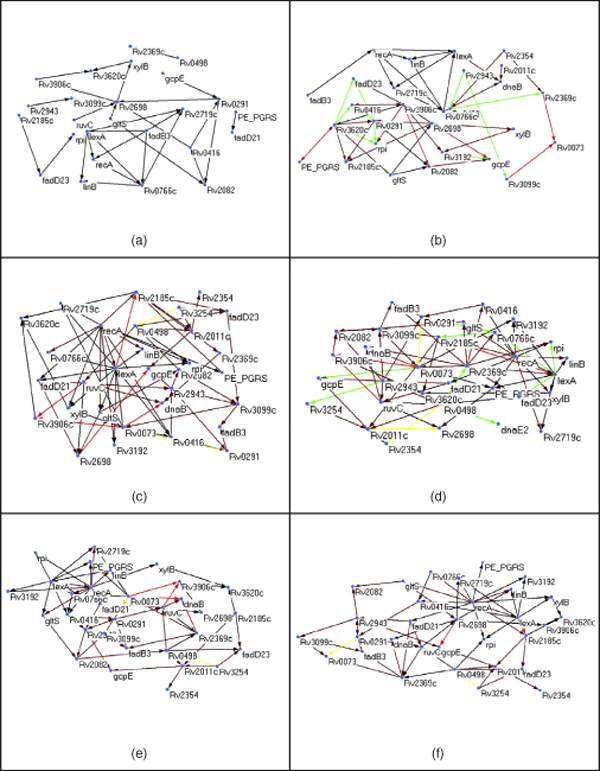
**Time-delayed interactions in predicted network of 32 genes**. Time-delayed interactions in predicted network of 32 genes (a) DBN network, (b) HDBN network, (c) Skip-chain network, (d) Skip-chain network with Gibbs prior, (e) Skip-chain network with Dirichlet prior, (f) Skip-chain network with Gibbs and Dirichlet prior.

The earlier network of 16 interactions predicted using correlations is shown in Fig. 2(a). It can be seen we compare well with this network. Fig. 2(b) gives the color code. Fig. 3 and 4 are predicted networks by our algorithm. The prediction using the first-order DBN and third-order HDBN are shown in Fig. 3(a) and 3(b). The HDBN detects lexA-linB as a time-delayed interaction over 2 hrs. Some interactions are correctly detected by HDBN over DBN, for example: ruvC-fadD23. The skip-chain model in Fig. 3(f) detects inhibition of ruvC by recA even at 4 hrs. This is biologically plausible as the DNA repair in the genome spans over 10 hrs. The interaction between lexA-fadD21 is also detected by the skip-chain.

The presence of hubs or single genes regulating several other genes are also seen in the network. These networks can buffer environmental variations. It can be seen that a small number of transcription factors (TF) regulate the rest of the repair system. At the same time the in-degree is low, as each gene is regulated by just one TF. RecA causes inactivation of lexA which suppresses DNA repair genes. We observe binding of recA(DNA repair) to dnaB(DNA replication) helicase. RecA also activates linB which causes dehalogenation needed for transformation events in dna repair. The Fadd genes initiate apoptosis and are also required for cell-wall formation.

The second dataset of 32 genes indicated that our method is good for identifying core genes (Fig. 4). RecA and lexA are shown to be critical hub by both DBN and HDBN. The HDBN showed several time-delayed interactions at 2 and 4 hrs. The skip-chain gave a fewer interactions though it also showed interactions at 6 hrs. Use of prior gives better networks with few hubs in Fig. 4(f). They could detect new hubs like ruvC, fadD21 and fadD23.

## Discussion and conclusion

An organism responds to changes in its environment by altering the level of expression of critical genes. The virulence of *Mycobacterium tuberculosis *depends on the ability of the bacilli to switch between replicative (growth) and non-replicative (dormancy) states in response to host immunity. Different transcription networks are active at different stages of the response. The coordinated repression of genes are likely to contribute to survival by conserving energy and precursors under nutrient-limiting conditions and/or minimizing expression of potential antigens.

*M. tuberculosis *is known to have an unusally long period of 10 hrs for the DNA replication fork to traverse the chromosome. Our results showed several interactions at 4 hrs in the DNA repair pathway. An order-4 HDBN with skip-chain dependencies was shown to outperform ordinary HDBN's. For genes to interact they both have to be upregulated. We use this property to select events where a pair of genes are both upregulated at similar or delayed time points. It is well established that interacting genes have correlated expression patterns. To this end, we add the interactions at non-consecutive time points. This is because a DBN assumes a first-order network and is not able to model complex time-delayed interactions. We assumed that all interactions had equal priors. However our method is able to distinguish between short- and long-term interactions and hence allow us to make a better judgement on DNA repair.

To include time-delays, we used a skip-chain model. The Viterbi shortest path allowed us to choose between time delayed interactions of two genes of same and different time delays. This lets us identify the best interaction information from the dataset. By using a single parent Viterbi path to model the upregulated events, we were able to focus on special cases in the DBN. This significantly reduces the search space for the GA. Our search is however constrained by various parameters like MI and number of parents.

Skip-chain models address the difficulties of a DBN by easily incorporating overlapping input features. We also see that using approximate inference leads to lower total training time without loss in accuracy. The skip-chain BN is not an HDBN because usually different long-distance dependencies are used by skipping the intermediate time points. We proposed a method that can extract long distant regulations and demonstrated it on DNA repair of tuberculosis. Our approach may be useful for understanding complex gene regulation mechanisms.

Lastly, using priors gave us higher likelihood and improved the over-fitting in building the regulatory networks. The Dirichlet prior gave fewer hubs as compared to the Gibbs prior and gave a higher likelihood. The combination of the two priors gave us the best regulatory networks. We can see that the prediction with prior allows higher-orders of linear model aswell.

## Competing interests

The authors declare that they have no competing interests.

## Authors' contributions

I. Chaturvedi implemented the algorithm and wrote the initial draft. J. C. Rajapakse guided the project, and reformed later drafts of the manuscript. All authors read and approved the final manuscript.

## Note

Other papers from the meeting have been published as part of *BMC Bioinformatics* Volume 10 Supplement 15, 2009: Eighth International Conference on Bioinformatics (InCoB2009): Bioinformatics, available online at http://www.biomedcentral.com/1471-2105/10?issue=S15.

## References

[B1] Friedman N Murphy K Russell S Learning the Structure of Dynamic Probabilistic Networks Proceedings of the 14th Annual Conference on Uncertainty in Artificial Intelligence (UAI-98) 1998 139 14

[B2] Zhengzheng X Dan W Modeling Multiple Time Units Delayed Gene Regulatory Network Using Dynamic Bayesian Network Data Mining Workshops, 2006. ICDM Workshops 2006. Sixth IEEE International Conference on 2006 190 195

[B3] Chaturvedi I Rajapakse J Fusion of Gene Regulatory and Protein Interaction Networks Using Skip-Chain Models Pattern Recognition in Bioinformatics 2008 5265 Lecture Notes in Computer Science, Springer Berlin/Heidelberg 214 224

[B4] Galley M A Skip-Chain Conditional Random Field for Ranking Meeting Utterances by Importance Proceedings of the 2006 Conference on Empirical Methods in Natural Language Processing (EMNLP 2006) 2006 Sydney: Association for Computational Linguistics 364 372

[B5] Sutton C McCallum A Collective Segmentation and Labeling of Distant Entities in Information Extraction Presented at ICML 2004 Workshop on Statistical Relational Learning 2004

[B6] Fink GA service SO Markov Models for Pattern Recognition From Theory to Applications 2008

[B7] Hao T Huang TS Improved Graphical Model for Audiovisual Object Tracking Multimedia and Expo, 2006 IEEE International Conference on 2006 997 1000

[B8] Shuanhu B Haizhou L Bayesian Learning of N-Gram Statistical Language Modeling Acoustics, Speech and Signal Processing, 2006. ICASSP 2006 Proceedings. 2006 IEEE International Conference on 2006 1 I-I

[B9] Shmulevich I Zhang W Binary analysis and optimization-based normalization of gene expression data Bioinformatics 18 4 555 65 10.1093/bioinformatics/18.4.555 12016053

[B10] Gebert J Motameny S Faigle U Forst CV Schrader R Identifying Genes of Gene Regulatory Networks Using Formal Concept Analysis Journal of Computational Biology 2008 15 2 185 194 10.1089/cmb.2007.0107 18312149

[B11] Francis RB Michael IJ Kernel independent component analysis J Mach Learn Res 2003 3 1 48 10.1162/153244303768966085

[B12] Suri RE Application of independent component analysis to microarray data Integration of Knowledge Intensive Multi-Agent Systems, 2003. International Conference on 2003 375 378

